# Immune control of an SIV challenge by a heterolgous and direct mucosal vaccination regimen in rhesus monkeys

**DOI:** 10.1186/1742-4690-9-S2-O3

**Published:** 2012-09-13

**Authors:** C Sun, Z Chen, X Tang, Y Zhang, L Feng, Y Du, L Xiao, L Liu, W Zhu, L Chen, L Zhang

**Affiliations:** 1 Guangzhou Institute of Biomedicine and Health, CAS, Guangzhou, China; 2 AIDS Institute, LKS Faculty of Medicine, University of Hong Kong, China; 3 AIDS Institute, LKS Faculty of Medicine, University of Hong Kong, China; 4 Guangzhou Institute of Biomedicine and Health, CAS, China; 5 Guangzhou Institute of Biomedicine and Health, CAS, China; 6 AIDS Institute, LKS Faulty of Medicine, University of Hong Kong, China; 7 AIDS Research Center, Chinese Academy of Medical Sciences, China; 8 Comprehensive AIDS Research Center, Tsinghua University, Beijing, China

## Background

The mucosal surface is the major route for HIV-1 transmission, yet a safe and effective AIDS vaccine through direct mucosal immunization remains elusive.

## Methods

Here, we report a novel vaccination regimen consisting of a mucosal prime with replication-competent vaccinia Tiantan rMVTTSIVgpe and an intramuscular boost with non-replicating rAd5SIVgpe expressing SIV Gag, Pol and Env. Twenty Chinese rhesus macaques were used to evaluate its safety, immunogenicity and protective potential.

## Results

Compared with three control groups, the rMVTTSIVgpe-rAd5SIVgpe regimen elicited robust cellular immune responses with enhanced magnitude, sustainability and polyfunctionality, and higher titers of neutralizing antibodies against SIVmac1A11. Moreover, one rMVTTSIVgpe-rAd5SIVgpe vaccinated animal was fully protected, while the rest demonstrated 1.74-log and 1.2-log reductions in peak and set-point viral loads upon intrarectal challenge with a high dose (5x105 TCID50/animal) of a pathogenic and neutralization-resistant SIVmac239. Importantly, the rMVTTSIVgpe-rAd5SIVgpe vaccinated animals remained healthy up to 850 days post-challenge, while the majority (~75%) of controls progressed to simian AIDS. The protective effect was found to correlate with SIV-specific CD8+ T cell ELIspot responses against Gag and Pol, but not Env.

## Conclusion

Our findings indicate that vaccine strategy engaging the mucosal surface from the beginning of vaccination may provide protective immunity against HIV-1 infection in humans.

